# The use of four K‐wires does not lead to a reduction of the MPTA in the context of a one‐dimensional tibial deflection correction of patients with ACL re‐rupture and pathologically increased tibial slope

**DOI:** 10.1002/jeo2.70145

**Published:** 2025-01-26

**Authors:** Christian Arras, Alexander Korthaus, Jannik Frings, Markus T. Berninger, Hendrik Fahlbusch, Karl‐Heinz Frosch, Tobias Drenck, Ralph Akoto, Matthias Krause

**Affiliations:** ^1^ Department of Trauma and Orthopaedic Surgery University Medical Center Hamburg‐Eppendorf Hamburg Germany; ^2^ Department of Trauma Surgery, Orthopaedics and Sports Traumatology BG Hospital Hamburg Hamburg Germany; ^3^ Department of Orthopaedic Surgery, Trauma Surgery and Sports Medicine Cologne Merheim Medical Center (Witten/Herdecke University) Cologne Germany

**Keywords:** ACL revision surgery, anterior tibial closing wedge osteotomy, medial proximal tibial angle (MPTA), posterior tibial slope (PTS)

## Abstract

**Purpose:**

Anterior tibial closing wedge osteotomy (ATCWO) has been shown to significantly reduce failure rates of revision anterior cruciate ligament (ACL) reconstructions in patients with a posterior tibial slope (PTS) ≥12°. Recent findings suggest a slight but significant reduction of the medial proximal tibial angle (MPTA) resulting in a varus knee where the sagittal osteotomy plane is based on a total of two guide wires defining the osteotomy wedge without respecting the frontal plane. We hypothesize that the placement of a total of four guide wires intraoperatively can reduce the influence on the MPTA.

**Methods:**

This study retrospectively reports on a two‐centre series of 42 ATCWOs for PTS correction between January 2022 and December 2023 at two clinical centres. A total of four guide wires were placed based on a true lateral intraoperative view of the tibia, with two positioned each at the cranial and at the caudal pole of the osteotomy wedge, serving as guides for the saw to create the osteotomy, with careful attention to ensuring that the proximal and distal K‐Wires were placed parallel to each other. A retrospective analysis was conducted by examining true lateral and anteroposterior radiographs to identify changes in sagittal and coronal plane alignment.

**Results:**

The study included 19 women and 23 men, with a mean age of 29.7 ± 8.6 years with first‐time ACL revision surgery and a minimum PTS of ≥12°. PTS decreased significantly from 14.5 ± 2.8° preoperatively to 8.2 ± 1.9° post‐operatively (*p* < 0.001). The aMPTA demonstrated no significant difference between preoperative (mean aMPTA: 86.9 ± 2.1°) and post‐operative (mean aMPTA: 86.6 ± 1.9°) measurements (*p* > 0.05).

**Conclusion:**

With our technique of placing four guide wires to achieve precise guidance during the insertion of the osteotomy wedge, there is no substantial impact on the aMPTA during slope correction.

**Level of Evidence:**

Level IV.

**Abbreviations**
a.p.: anterior‐posterior
ACL: anterior cruciate ligament
ACLR: anterior cruciate ligament reconstruction
aMPTA: anatomical medial proximal tibial angle
ATCWO: anterior tibial closing wedge osteotomy
ICC: intraclass correlation coefficient
MPTA: medial proximal tibial angle
OA: osteoarthritis
PAA: posterior anatomical axis
PCL: posterior cruciate ligament
PTS: posterior tibial slope
ROM: range of motion
sMCL: superficial medial collateral ligament

## INTRODUCTION

Increased posterior tibial slope (PTS) has been identified as a risk factor predisposing individuals to primary anterior cruciate ligament (ACL) injury and graft failure after ACL reconstruction (ACLR). ACLR failure rates are significantly increased in patients with a PTS ≥ 12° [10]. Anterior tibial closing wedge osteotomy (ATCWO) has been shown to significantly reduce revision ACL reconstruction failure rates in high‐risk patients with PTS ≥ 12° [1, 3, 21]. However, leg alignment in the coronal plane has also been shown to influence ACL tension. Valgus malalignment at 115% of the weight‐bearing line results in almost double the ACL graft forces [13]. However, more commonly, a 5° varus malalignment increases ACL stress by up to 264% at 30° of knee flexion, especially putting the ACLR at risk for secondary and, more importantly, tertiary ACLR failure [7, 19]. Hence, inadvertent changes in the coronal plane should be avoided or at least anticipated during ATCWO.

Recently, the influence of ATCWO on coronal plane changes has been debated, but different techniques have been described. While supratubercular ATCWO was not associated with clinically relevant changes in the mechanical medial proximal tibial angle (MPTA), infratubercular ATCWO significantly decreased the MPTA by an average of 1.3 ± 1.5° depending on the wedge seizure [3, 12].

In addition to its influence on ACL stress, varus‐producing PTS correction has significant clinical importance as frontal misalignment may have long‐term adverse effects on the patient, such as the early onset of medial‐sided osteoarthritis (OA), which is common in ACL‐deficient knees [2, 5]. In recent studies, the height of the osteotomy wedge is defined with two converging wires [6, 12, 18]. To maintain coronal alignment, the sagittal osteotomy cut must take into account the medially descending MPTA with respect to the triangular shape of the proximal tibia. Therefore, placing four instead of two converging K‐wires may result in fewer coronal axis changes compared to literature findings. Our hypothesis is that MPTA alterations can be avoided by increasing the number of guide wires to four, thereby addressing the frontal plane. We aim to demonstrate that the intraoperative placement of a total of four guide wires can prevent any influence on the MPTA by simultaneously respecting both the sagittal and frontal planes.

## METHODS

### Study population

This study retrospectively reports on a two‐centre series of 42 ATCWOs for PTS correction between January 2022 and December 2023 at two clinical centres.

Specifically, the analysis focused on patients who underwent a slope correction osteotomy followed by autograft revision ACLR in a two‐stage procedure. This retrospective case series was limited to patients with ACLR failure and a PTS ≥ 12 degrees. All patients with an indication for an isolated one‐dimensional slope correction between January 2022 and December 2023 were included. Patients undergoing combined osteotomies correction of the leg axis in two or more planes, post‐traumatic deformities after tibial plateau or proximal tibial fracture as well as patients with congenital malformations of the axial skeleton were excluded. This exclusion criterion was established to isolate the effect of the slope correction osteotomy on the anatomical MPTA (aMPTA). Malrotated preoperative sagittal radiographs with an overlap of >7 mm between the medial and lateral posterior tibial condyles were also excluded. All cases included had true lateral and anteroposterior radiographs before and after surgery. Post‐operative radiographs were taken 2 days after the index surgery.

### Measurement and definition of PTS and aMPTA

For the measurements of the PTS and the MPTA, the available knee radiographs as well as long‐leg radiographs were used. The PTS was measured as previously described [4, 23, 25]. On a true lateral radiograph of the tibia, the PTS is determined quantitatively by first establishing the posterior anatomical axis (PAA) of the tibia. The PAA is defined as a line intersecting the midpoint of the tibial shaft at two defined points, specifically 5 cm and 15 cm inferior to the tibial plateau's joint line. Subsequently, the PTS is defined as the angle formed by the intersection of a line tangential to the medial tibial plateau and a line orthogonal to the PAA [4, 12].

The anatomical medial proximal angle was also measured as previously described [17]. First, the proximal anatomical tibial axis must be defined by the identification of two midpoints along the tibial cortex. The first midpoint was placed 10 cm inferior to the joint line, while the second midpoint was placed at the most distal point allowed by the radiograph, but no more than 20 cm distal to the joint line [12, 17]. The aMPTA is the medial angle between the proximal anatomical tibial axis and the tibial plateau in the coronal view.

**Figure 1:**
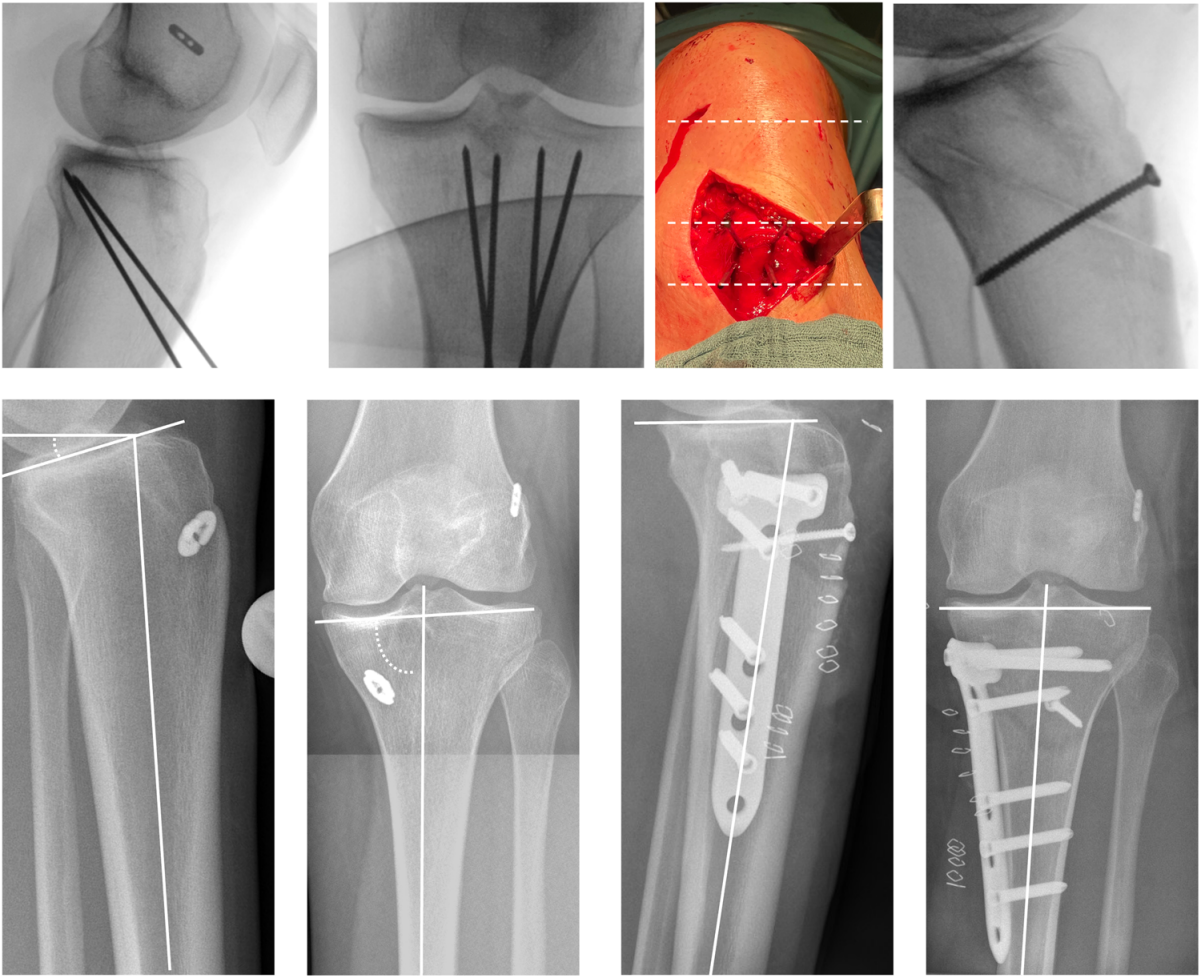
X‐ray of 18‐year‐old female, initial ACL rupture with partial medial meniscus loss following a bicycle fall. (a) Intraoperative lateral view of the proximal tibia with four inserted guide wires for marking the osteotomy, the proximal and distal being completely parallel to each other. (b) Intraoperative a.p. view of the proximal tibia with four inserted guide wires for marking the osteotomy. (c) Intraoperative image showing the K‐wires. The dashed lines show the approximate height of the joint space when viewed from proximal to distal, the following proximal two K‐wires and the distal two K‐wires, which parallel to the joint space. (d) Intraoperative lateral view with inserted screw after osteotomy. (e, f) Preoperative measure of PTS (15°) in lateral (d) and aMPTA (84.2°) in a.p. (e) view. (g, h) Post‐operative measure of PTS (7.7°) in lateral (f) and aMPTA (84.4°) in a.p. (g) view. ACL, anterior cruciate ligament; aMPTA, anatomical medial proximal tibial angle; a.p., anterior‐posterior; PTS, posterior tibial slope.

All radiographs were analyzed by two fellowship‐trained orthopaedic surgeons (Figure 1). In addition, a test for intra‐ and inter‐observer variability was performed, with intra‐observer measurements taken at 6‐week intervals.

### Surgical technique

Preoperative planning of the osteotomy was performed using the Sentra two‐dimensional planning system software (Sentra), aiming for a PTS of 8°. Based on a true lateral view, a proximal tibia of at least 25 cm in length was used. The proximal aspect of the osteotomy was defined as at least 6 cm distal to the proximal rim of the tibial plateau, starting at the level of the joint line. The hinge point of the osteotomy was directed towards the centre of the tibial bony insertion of the posterior cruciate ligament (PCL).

During surgery, the patient was positioned to allow for true lateral and true anterior‐posterior (a.p.) views of the proximal tibia using the intraoperative imaging device. Care was taken to ensure that the two plateaus also overlapped, and that the fibula tended to be dorsal to the tibia in order to obtain a true lateral radiograph. The surgical approach began approximately 2 cm medial to the tuberosity and 5 cm distal to the joint line with a skin incision of approximately 5 cm in length. A blunt dissection was performed down to the fascia of the sartorius muscle, and an incision was made just above the hamstring tendons if they were still present. The superficial medial collateral ligament (sMCL) was not released as the osteotomy was performed below its anatomical attachment [9]. Based on a true lateral view, two parallel 1.8 mm guide wires were oriented towards the posterior hinge located at the lower tibial PCL attachment (Figure 1a). Two additional 1.8 mm guide wires were placed parallel towards the posterior hinge more distally, depending on the planned wedge height (Figure 1b). Special care was taken to ensure that all four wires were parallel to the joint line in order to avoid coronal malalignment (Figure 1a–c). Similarly, in the true lateral view, the proximal and distal wires are aligned in parallel to ensure that the hinge runs parallel to the joint line. The osteotomy was performed using an oscillating saw, paying particular attention to the medial cortex in order to resect the wedge sufficiently and also to spare the distal remnant fibres of the sMCL. The osteotomy was completed by using chisels to avoid posterior hinge fractures. Subsequently, the bone wedge was removed, the osteotomy closed in full extension and compressed with one or two compression screws (Figure 1d). Finally, an anteromedial fixed‐angle plate was placed and fixed with fixed‐angle screws only (Figure 1g,h). Compared to the preoperative measurement, a corrected PTS is shown without a significant change in the aMPTA (Figure 1e–h).

### Statistical analysis

Power analysis was performed using the ‘Means: Difference from constant (one sample case)’ statistical *t* test (*p* ≤ 0.05). The retrospective analysis with the cohort of 42 patients revealed a power of 0.93.

Descriptive statistics are presented as means with their standard deviations. The paired Student's *t* test was used to assess mean differences between preoperative and post‐operative scores for parameters that followed a normal distribution.

For intra‐ and interobserver variability the intraclass correlation coefficient (ICC) was chosen because of its ability to quantify the strength and direction of linear relationships between the variables [8, 20].

Statistical evaluations were performed using IBM SPSS Statistics, version 29.0.2, with a *p* < 0.05 considered indicative of statistical significance.

## RESULTS

The study included 19 women and 23 men, with a mean age of 29.7 ± 8.6 years with first‐time ACL revision surgery and a minimum PTS of ≥12° (Table 1). PTS exhibited a significant decrease from 14.5 ± 2.8° preoperatively to 8.2 ± 1.9° post‐operatively (*p* < 0.001, Table 2). The aMPTA demonstrated no significant difference between preoperative (mean aMPTA: 86.9 ± 2.1°) and post‐operative (mean aMPTA: 86.6 ± 1.9°) measurements (Table 2).

**Table 1:** Study group.

No. of patients	42
Age, years, mean ± SD (range)	29.7 ± 8.6 (17–49)
Sex (% of total)	
Male	23 (54.8%)
Female	19 (45.2%)

Abbreviation: SD, standard deviation.

**Table 2:** Radiographic measurements.

Posterior tibial slope (PTS) (°)	
Preoperative	14.5 ± 2.8°, 12–21.7° (mean, SD, range)
Post‐operative	8.2 ± 1.9°, 5–12.8°
Average PTS change	6.2 ± 1.9°
Anatomical medial proximal tibial angle (°)	
Preoperative	86.9 ± 2.1°, 83.1–92.1°
Post‐operative	86.6 ± 1.9°, 82–90°

Abbreviation: SD, standard deviation.

The overall complication rate was low (Table 3). There were no instances of late surgical site infections, hinge fractures or pseudoarthrosis/delayed union. Two patients suffered a deep vein thrombosis, and nine patients described hardware irritation at the late check‐up prior to revision ACL surgery.

**Table 3:** Short‐term outcomes and complications after anterior tibial closing wedge osteotomy.

Outcomes and complications	*N* (%)
Preoperative ROM (*n* = 42)	
Hyperextension 0–5°	1
Hyperextension >5°	1
Flexion <100°	0
Extension <0°	4
Post‐operative ROM, early check‐up (*n* = 42)	
Hyperextension 0–5°	0
Hyperextension >5°	0
Flexion <100°	3
Extension <0°	11
Post‐operative ROM, late check‐up (*n* = 42)	
Hyperextension 0–5°	0
Hyperextension >5°	1
Flexion <100°	0
Extension <0°	1
Complications	
Late surgical‐site infection	0
Hardware irritation at late check‐up	9
Hinge fracture	0
Pseudoarthrosis/delayed union	0
Other complications	2 deep vein thrombosis

Abbreviation: ROM, range of motion.

The intra‐ and interobserver ICCs for the measurement of aMPTA and PTS were good to excellent (good defined as 0.75 ≤ ICC < 0.9, excellent defined as ICC > 0.9). The intraobserver ICC for the preoperative aMPTA was 0.95 and for the post‐operative aMPTA 0.84. The interobserver ICC stated an ICC of 0.92 for the preoperative and 0.86 for the post‐operative aMPTA. For both pre‐ and post‐operative PTS the intra‐ and interobserver ICC were >0.9.

Intraoperative examination of the knee joint during slope correction surgery and ACL revision surgery showed no difference in the medial stability of the knee joint in the patient collective.

## DISCUSSION

The findings of this study suggest that frontal plane alterations during ATCWO can be avoided using four K‐wires instead of two. In our study, we investigated the effect of considering the frontal plane during ATCWO for slope correction in cases of pathologically increased PTS in ACL revision surgery.

In the present study, we demonstrated that by placing four guide wires to define the osteotomy wedge for ATCWO, there was no significant difference in the post‐operative aMPTA compared to the preoperative aMPTA.

This is in contrast to a recently published study by Mayer et al. who showed a change in MPTA of 1.3 ± 1.5° [12]. In another study, Weiler et al. also showed a change in MPTA of 1.1° with ATCWO slope correction [24]. Although a clear static significance could not be determined, these studies refer to technical descriptions of the slope correction, at least two of which included a two K‐wire marking of the osteotomy. In these cases, the two K‐wires were inserted vertically relative to each other, marking the width of the osteotomy and taking into account the sagittal plane.

Although these alterations may seem small, they are still of great clinical importance, as an increasing varus deformity, even in the 1–2° range, has a major impact on ACLR survival and unilateral joint degeneration [7, 19]. A varus deformity of 5° and greater increases the tension and force on the ACL by up to 264%, which has been described as a significant factor contributing to ACL re‐ruptures [7, 19]. In addition to increased tension on the ACL, an increasing varus deformity also increases the risk of unilateral cartilage damage, particularly in the medial joint space [15]. Considering that many ACL injuries are associated with medial meniscus damage and subsequent medial cartilage damage, even a minimal varus deformity with deviations of 1.3° to a maximum of 3° after slope correction plays a crucial role [22].

In the present study, we hypothesize that the use of four instead of two K‐wires will result in greater precision of the osteotomy wedge, thereby preventing alteration of the coronal plane. Regardless of the size of the osteotomy wedge and regardless of the initial aMPTA, there was no change in the aMPTA after PTS correction as long as the osteotomy and posterior hinge were parallel to the joint line [12].

**Figure 2:**
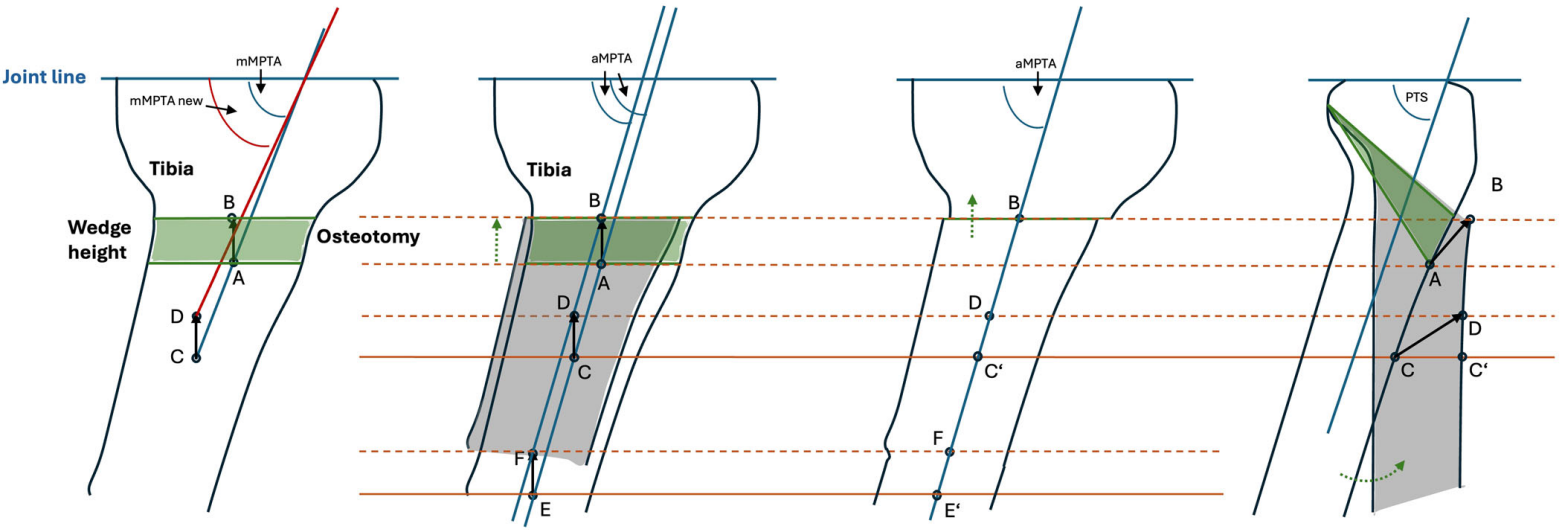
Geometric model of an anterior tibial closing wedge osteotomy (ATCWO) in coronal and sagittal view with a medial proximal tibial angle (MPTA) ≠ 90°. (a) Schematic representation of the model by Mayer et al. for ATCWO at MPTA ≠ 90°. The line of the new proximal tibia axis does not cross Point B. (b) Illustration of the proximal tibia according to our model with parallel displacement of the proximal tibial axis without changing the anatomical MPTA (aMPTA) post‐operatively. The grey area shows the position of the tibia distal to the osteotomy after closing of the wedge. The proximal tibial axis originally runs through Points C and E and forms the aMPTA at the intersection with the joint line. Point A is defined as the intersection of the proximal tibial axis with the distal osteotomy line. After closing of the Wedge, the proximal tibia axis now also crosses Point B. (c) Illustration following the completion of ATCWO. The solid red lines represent the height of the two centres of the tibia used to determine the proximal tibial axis. (d) Sagittal view. The tibia distal to the osteotomy rotates anteriorly along a circular path during the closure of the osteotomy.

There are several aspects to consider when compared to previous explanations: First, the Mayer et al. [12] geometric model of a PTS deflection osteotomy has been adapted to reflect a case with a proximal anatomical tibial axis and aMPTA less than 90° (Figure 2). It should be emphasized that based on pre‐ and post‐operative short a.p. radiographs of the proximal tibia, the aMPTA is determined by connecting the midpoints of both tibial cortices 10 cm below the joint line and as far distally as the radiograph allows [12]. Hence, with respect to previous theoretical considerations, we agree that Point A moves to Point B and Point C to Point D after the osteotomy. But the proximal midpoint is placed 10 cm inferior to the joint line, and therefore below the osteotomy, which itself is located approximately 6–7 cm below the joint line (Figure 2a). After PTS correction, Point A becomes Point B, Point C becomes Point D and Point E becomes Point F (Figure 2b,c). Hence, based on the measuring technique by Petersen et al. [16]. The pre‐ and post‐osteotomy anatomical axes shift parallel to each other because the fixed points for measurement in the tibial shaft are distal to the osteotomy site (Figure 2b). Figure 2c shows the aMPTA measurement after wedge closure, which is not altered as long as the osteotomy or posterior hinge is placed strictly parallel to the existing articular surfaces. However, the aMPTA after osteotomy closure, as described above, corresponds to the angle between the anatomical proximal tibial axis and the tibial plateau, not the mechanical axis typically used in the measurement of long‐leg radiographs. Therefore, and second, the second osteotomy rule defined by Paley needs to be respected [16]. As the MPTA on a long‐leg radiograph always runs through the centre of the tibial spine and the angulation correction axis and osteotomy are at different levels, angulation and translation should be observed. Hence, a minor decrease in MPTA should be observed, which we did not. Angulation of the osteotomy plane may also affect the MPTA [14]. Most importantly, we were able to show that the hinge should be placed strictly parallel to the joint line and that the use of at least two parallel wires per plane improves reliability in ensuring that the osteotomy can be cut parallel to the joint surfaces.

Most interestingly, in supratubercular ATCWO, the use of four K‐wires resulted in a smaller but still significant mean decrease in MPTA of 0.95° in 68 osteotomies [3]. Although these data seem to contradict our hypothesis of an increased wedge accuracy in ATCWO, the supratubercular is a more demanding osteotomy technique as the preservation of the patellar tendon as well as the wider tibial plateau may alter the precision of the saw precision and influence the parallelism of both osteotomy cuts [11]. Other surgical peculiarities could include a lack of resection in the lateral cortex, an increased medial cortical resection due to harder bone stock resulting in an asymmetric osteotomy gap or a unilateral directional osteosynthetic compression of the osteotomy gap. However, in our opinion, four K‐wires improve the guidance of the saw cut and in addition to leaving out medial osteotomy gap compression thus lead to an isolated correction of the slope.

## LIMITATIONS

In isolated cases, the pre‐ and post‐operative radiographs, both in the anteroposterior and lateral views, were not accurately aligned, which could lead to measurement inaccuracies.

Long‐term clinical and radiological outcomes after slope correction have not been investigated; for example, it would be interesting to investigate how the leg axis appears on a full‐leg standing radiograph after the patient is allowed to bear full weight. Patient‐specific factors such as comorbidities or anatomical characteristics may have an additional effect that was not considered in our study.

The study is retrospective and does not include a control group (e.g., match‐pair analysis with the two K‐wires technique), which would prove a direct correlation between the described change in MPTA and the surgical technique.

## CONCLUSION

With our technique of intraoperative placement of four guide wires to provide precise guidance during the insertion of the osteotomy wedge, there is no significant impact on the MPTA during slope correction.

The study presents a method that appears to resolve the previously described problem of systematic varus deformity following slope correction and is therefore considered highly relevant by the authors.

## AUTHOR CONTRIBUTIONS

*Study conceptualization*: Christian Arras, Alexander Korthaus, Karl‐Heinz Frosch and Matthias Krause. *Methodology*: Christian Arras, Alexander Korthaus, Heinz Frosch and Matthias Krause. *Data curation*: Christian Arras, Alexander Korthaus, Jannik Frings, Tobias Drenck and Markus T. Berninger. *Validation*: All authors. *Formal analysis*: Christian Arras, Alexander Korthaus, Markus T. Berninger, Jannik Frings and Matthias Krause. *Resources*: Christian Arras, Alexander Korthaus, Jannik Frings and Tobias Drenck. *Manuscript draft preparation*: Christian Arras, Alexander Korthaus, Ralph Akoto and Matthias Krause. *Review and editing*: All authors. *Supervision*: Karl‐Heinz Frosch, Ralph Akoto and Matthias Krause. *Project administration*: Matthias Krause.

## CONFLICT OF INTEREST STATEMENT

The authors declare no conflicts of interest.

## ETHICS STATEMENT

The research project underwent an ethical review by the Ethics Committee and a positive statement (2024‐101279‐BO‐ff) is available. Informed consent was obtained from all participants in the study.

## Data Availability

The raw data are available upon reasonable request from the corresponding author.
